# Clinical outcomes of percutaneous microwave ablation for pulmonary oligometastases from hepatocellular carcinoma: a retrospective, multicenter study

**DOI:** 10.1186/s40644-024-00679-7

**Published:** 2024-03-04

**Authors:** Gang Wang, Zhigang Wei, Feihang Wang, Xiaoying Han, Haipeng Jia, Danyang Zhao, Chunhai Li, Lingxiao Liu, Xia Yang, Xin Ye

**Affiliations:** 1 Department of Oncology, Shandong Key Laboratory of Rheumatic Disease and Translational Medicine, The First Affiliated Hospital of Shandong First Medical University & Shandong Provincial Qianfoshan Hospital, Shandong Lung Cancer Institute, 16766 Jingshi Road, 250021 Jinan, Shandong China; 2 Department of Interventional Radiology, Zhongshan Hospital, Fudan University, 200032 Shanghai, China; 3 National Clinical Research Center for Interventional Medicine, Zhongshan Hospital, Fudan University, Shanghai, China; 4 Shanghai Institute of Medical Imaging, Fudan University, Shanghai, China; 5 Department of Oncology, Shandong Provincial Hospital Affiliated to Shandong First Medical University, 250014 Jinan, Shandong China; 6 Department of Radiology, Qilu Hospital of Shandong University, 250012 Jinan, China

**Keywords:** Pulmonary oligometastases, Hepatocellular carcinoma, Microwave ablation, Multicenter study

## Abstract

**Background:**

Pulmonary oligometastases are common in hepatocellular carcinoma (HCC), however, the existing therapeutic options have several limitations. This study aimed to assess the safety and efficacy of microwave ablation (MWA) in the treatment of HCC-originating pulmonary oligometastases.

**Methods:**

A total of 83 patients, comprising 73 males and 10 females with a median age of 57 years, who had pulmonary oligometastases from HCC, underwent MWA treatment at four different medical institutions. Inclusion criteria for patients involved having primary HCC under control and having less than three oligometastases with a maximum diameter of ≤ 5 cm in the unilateral lung or less than five oligometastases with a maximum diameter of ≤ 3 cm in the bilateral lung. A total of 147 tumors were treated with MWA over 116 sessions. The primary endpoints assessed included technical success, treatment efficacy, and local progression rate, while secondary endpoints encompassed complications, clinical outcomes, overall survival (OS), local progression-free survival (LPFS), and prognostic factors.

**Results:**

The technical success rate for MWA was 100% (116/116 sessions), and the treatment efficacy rate was 82.3% (121/147 tumors). Six months after MWA, the local progression rate was 23.1% (18/147 tumors). Complications were observed in 10.3% (major) and 47.4% (minor) of the 116 sessions, with no cases of ablation-related deaths. The median follow-up period was 21.6 months (range: 5.7–87.8 months). Median OS was 22.0 months, and the 1-, 2-, and 3-year OS rates were 82.6%, 44.5%, and 25.2%, respectively. Median LPFS was 8.5 months. Multivariate Cox regression analysis identified α-fetoprotein (AFP) levels during initial diagnosis and the number of oligometastases as potential independent prognostic factors for OS (*p* = 0.017 and 0.045, respectively).

**Conclusion:**

Percutaneous MWA is a safe and effective treatment modality for pulmonary oligometastases originating from HCC.

## Introduction

Lungs are extremely common sites of metastatic spread in hepatocellular carcinoma (HCC) patients, accounting for approximately 39.5–53.8% of all extrahepatic metastases [1], which results in mortality and decreased overall survival (OS) in these patients [2]. Although pulmonary oligometastases have relatively good prognoses and can be potentially cured, the treatment is challenging and often involves multiple modalities, including systemic chemotherapy, immunotherapy, targeted therapies, stereotactic body radiotherapy, and surgery. Although surgical resection can improve the prognosis to some extent, not all patients are suitable for surgery [3, 4]. Image-guided thermal ablation (IGTA) might be an alternative treatment for such patients. IGTA is a precise, minimally invasive technique that is being increasingly used to treat lung tumors [5–7]. Microwave ablation (MWA), an IGTA technique, is also used to locally treat lung tumors [8–14]. However, the efficacy and safety of MWA in treating pulmonary oligometastases from HCC are poorly understood. In this retrospective, multicenter study, we investigated the effectiveness and safety of MWA in treating pulmonary oligometastases in HCC patients, which can potentially facilitate its future clinical applications.

## Patients and methods

### Patients

This study was conducted at four institutions, including Zhongshan Hospital affiliated with Fudan University, Shandong Provincial Hospital Affiliated to Shandong First Medical University, The First Affiliated Hospital of Shandong First Medical University & Shandong Provincial Qianfoshan Hospital and Qilu Hospital of Shandong University. Ethical approvals were obtained from all four institutions. We collected data from 83 HCC patients with pulmonary oligometastases who underwent MWA from June 2015 to October 2022. The inclusion criteria were as follows: (1) patients aged between 18 and 80 years who had not received other treatments except MWA for lesions; (2) showed no evidence of progression of residual or recurring liver disease before MWA; (3) ≤ 3 or ≤ 5 oligometastases in a single lung or bilateral lungs, respectively; (4) tumor diameter ≤ 5 cm; (5) unsuitable or refuse surgery; (6) the follow-up was more than three months; (7) Eastern Cooperative Oncology Group (ECOG) performance status ≤ 2; and 8) no abnormal coagulability (platelet count ≥ 100 × 10^9^/L). The exclusion criteria were as follows: (1) serious interstitial pulmonary disease; (2) regional lymph node or distant metastases after previous tumor treatment; (3) acute myocardial or acute cerebral infarction during the past 30 days; and (4) tumors located near critical structures such as major blood vessels or the heart. The patient and tumor characteristics are listed in Table 1.

**Table 1:** Clinical characteristics of the patients

Patients’ details	No.	Percentage (%)
**Sex**		
Male	73	88
Female	10	12
**Age (year)**		
< 60	55	66.3
≥ 60	28	33.7
**History of chronic illness**		
Hypertension	13	15.7
Diabetes	10	12
Coronary artery disease	3	3.6
Without chronic disease	57	68.7
**Liver cirrhosis**		
Yes	58	69.9
No	25	30.1
**No. of HCC foci**		
1	44	53
≥ 2	39	47
**Maximum diameter range of HCC foci (cm)**		
<5	74	89.2
≥ 5	9	10.8
**Treatment modalities of HCC**		
Resection only	10	12
TACE only	12	14.5
Comprehensive treatment	61	73.5
**AFP levels when oligometastases were diagnosed (ng/mL)**		
≥ 500	35	42.2
< 500	50	60.2
**Extents of oligometastases**		
Unilateral Pulmonary	82	55.8
Bilateral Pulmonary	65	44.2
**Maximum diameter range of lesions (mm)**		
Unilateral Pulmonary	4–27	
Bilateral Pulmonary	7–42	
**No. of lesion ablation**	116	
1	58	50
2	20	34.5
3	6	15.5

### Ablation procedures

Pre-procedure planning includes: (1) determining the “gross tumor region (GTR)”, including the location, size, shape, and its nearby critical anatomic structures; (2) selecting the appropriate body position and the puncture sites on the body; (3) determining the puncture path and “target‑skin distance”.

The patients were administered with local anesthesia (1% lidocaine) and preemptive analgesia (morphine plus flurbiprofen) or local anesthesia and sufentanil (0.25-1 µg/kg, injected intravenously) [15]. After anesthesia, the skin was cut at the puncture point and an antenna was inserted through the deeper tissue layers into the GTR as planned by ablation experts with at least five years of experience. MWA was performed after the cold circulating pipes and pump were connected to the MWA antenna and machine. The procedure was terminated when the post-ablation ground-glass opacity (GGO) surpassed the GTR by at least 5 mm [5, 16]. After this, the target tissue is defined as a *post-ablation tumor zone* (PTZ) [5]. After the procedure, the antenna was removed, and the wound was locally disinfected and bandaged.

### Follow-up imaging

All patients received a non-contrast chest computed tomography (CT) scan 24–48 h after the procedure to detect any related complications. The first chest contrast-enhanced CT was performed one month after the procedure, whereas the second one was performed after 3 months to observe any complications and to ensure complete ablation of local lesions. Thereafter, chest CT was performed every six months, mainly to detect relapses of local lesions, formation of scars, and presence of new lung lesions. An annual chest CT was performed after two years.

### Assessment of treatment efficacy

The primary endpoints were technical success, efficacy, and local recurrence rate. Technical success is defined as the tumor being treated according to the protocol and being completely ablated. During the follow-up (prospectively defined time point), efficacy was confirmed if the tumor was completely ablated. The local response was determined with the baseline of the lesions 4–6 weeks after the procedure and evaluated based on the following criteria (Table 2) [5]. If local progression or incomplete ablation is identified a month after the MWA, up to two sessions could be conducted.

**Table 2:** Efficacy evaluation criteria for ablation

Efficiency evaluation	Evaluation criteria
Complete ablation	Disappearance of the lesion
	Complete formation of the cavity
	Fibrosis of the lesion, which may be a scar
	Reduction or no change or enlargement of the solid nodule, but no signs of enhancement on enhanced CT scan and/or no metabolic activity of the PET/CT
	Pulmonary atelectasis, no signs of contrast enhancement on CT scan of the lesion within the atelectasis, and/or no metabolic activity of the PET/CT
Incomplete ablation	Incomplete cavity formation with partial solidity and enhanced CT scan with signs of enhancement and/or PET/CT with metabolic activity
	Partial fibrosis with contrast enhancement on CT scan around or at the edge of the fibrosis and/or PET/CT with metabolic activity
	Solid nodules with no change in size or increase in size with CT scan with signs of contrast enhancement and/or PET/CT with metabolic activity signs of intensification, and/or PET/CT with metabolic activity
	Tumor cells found on the biopsy
Local progression	10 mm lesion enlargement with irregular or internal enhancement on CT and significantly increased FDG uptake on PET/CT
	New local lesions with new signs of enhancement on CT and/or significantly increased FDG uptake on PET/CT

The secondary endpoints included clinical outcomes such as OS, local progression-free survival (LPFS), and other complications, and prognostic variables including sex, age, comorbid chronic diseases such as hypertension, diabetes, and coronary artery disease, α-fetoprotein (AFP) levels when oligometastases diagnosed, number of lesions and their maximum diameter range, and the extent of oligometastases. OS was defined as the interval from initial ablation to either death or the latest date when the patient was still alive. LPFS was defined as the interval from initial ablation to radiologic evidence of local tumor progression or the latest follow-up date. Tumor response was evaluated according to RECIST1.1. Complications are reported according to the classifications of the American Society of Interventional Radiology (SIR) criteria [17]. A major complication is an event leading to substantial morbidity and disability (e.g., unexpected loss of an organ), which increases the level of care, leading to hospital admission, or substantially lengthens the hospital stay (SIR classifications C–E). The complications also include any conditions requiring blood transfusion or interventional drainage. Any patient death within 30 days of IGTA should be addressed (SIR classification F). All other complications were considered minor.

### Statistical analysis

*SPSS V22.0* was used to analyze the data. The baseline and clinical characteristics, or the frequency of occurrence, were expressed as median values and ranges. Survival rates were analyzed by log-rank test and illustrated using Kaplan-Meier plots. The Cox proportional hazards were applied to conduct univariate and multivariate analyses to determine the factors associated with OS. The factors with a *p*-value of < 0.5 in the univariate analysis were included in the multivariate analysis. The final model was chosen based on the variables with *p*-values < 0.05 in the multivariate analysis. All statistical tests were two-tailed, and a *p*-value < 0.05 was considered statistically significant.

## Results

### Demographic and clinical characteristics

Table 1 shows the baseline demographic characteristics of the patients. We analyzed 83 patients, including 73 (88%) males and 10 (12%) females. The median age of the patients was 57 years (range: 23–72 years). All patients belong to stage C according to Barcelona clinic liver cancer (BCLC) staging system or stage IIIB according to China liver cancer (CNLC) staging system.

**Fig. 1:**
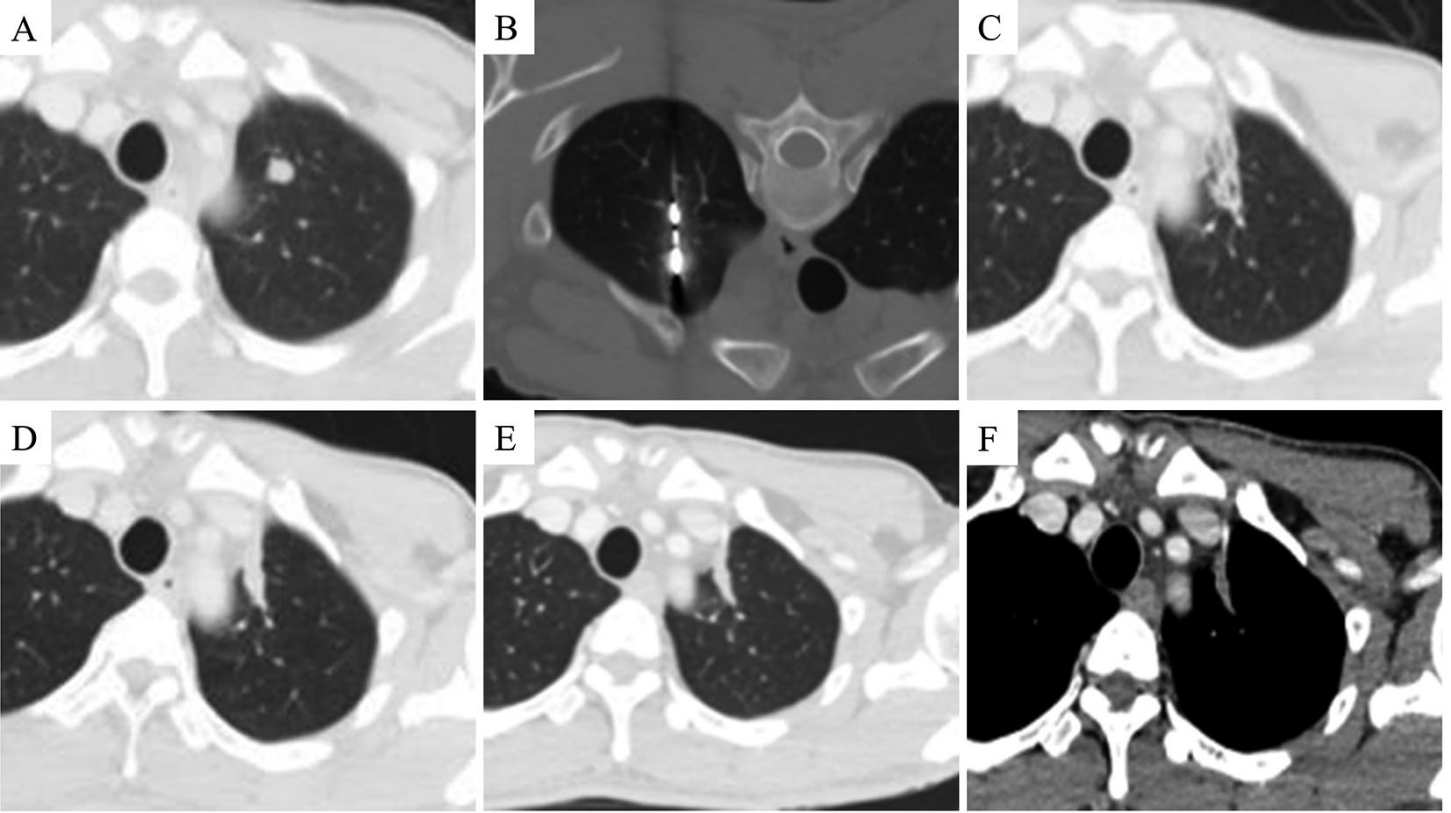
A 48-year-old male had a nodule in the upper left lung lobe for over four months, 16 months after primary hepatoma carcinoma surgery. **A** A 1.3 × 1.3 cm round nodule was seen in the left upper lung. **B** With the patient in the prone position and under local anesthesia with 2% lidocaine, CT-guided puncture MWA was performed. **C** A month after MWA, the lesion lost its original shape, and the surrounding exudate declined. **D** Six months after MWA, the lesion size was further reduced. **E**, **F** A year after MWA, the lesion shrank to a fibrous cord with no enhancement. The efficacy was assessed to achieve complete ablation

**Fig. 2:**
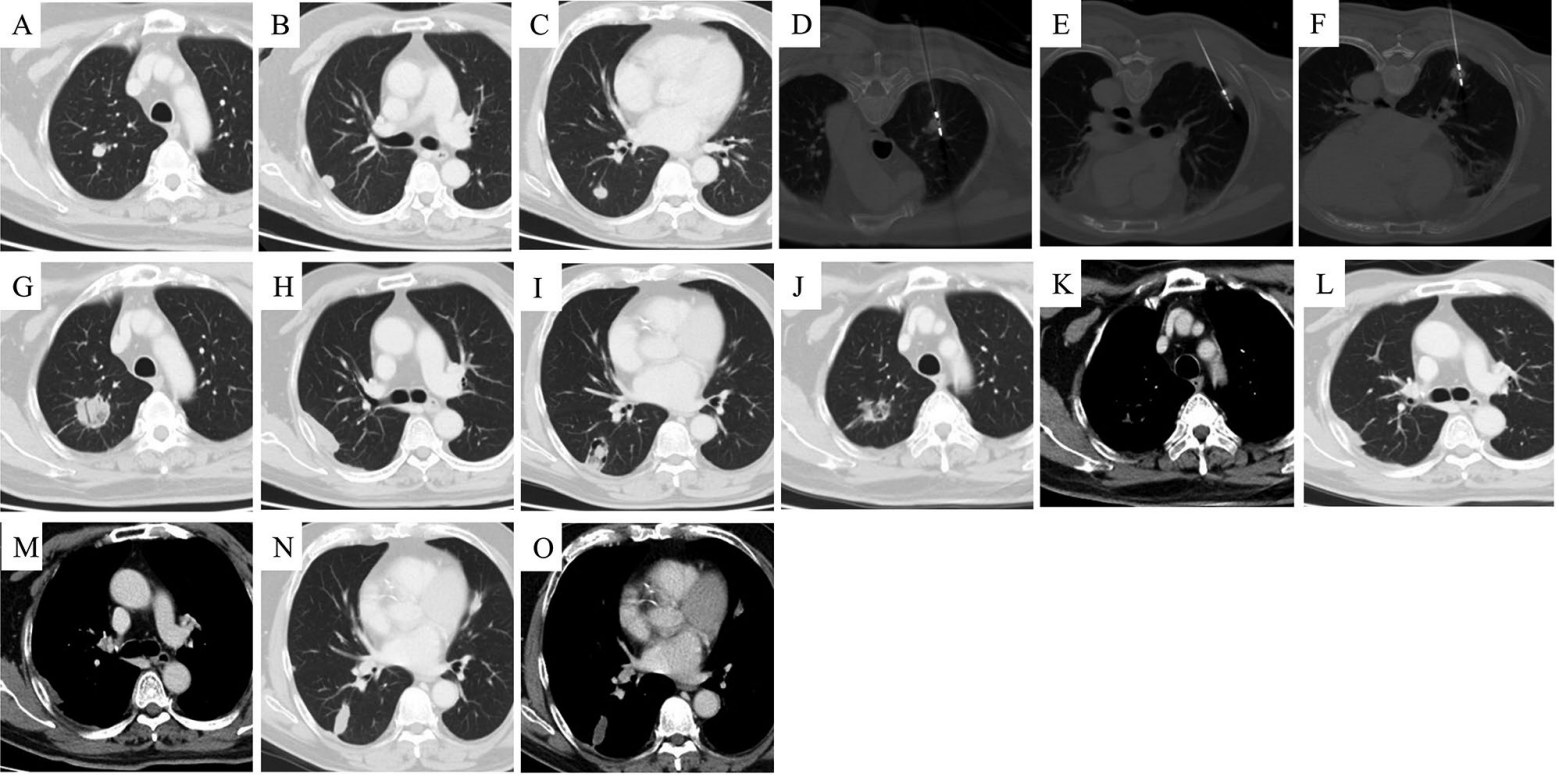
A 69-year-old male had three nodules in the right lung lobe for a month, detected nine months after primary hepatoma carcinoma surgery. **A** Nodule #1 (1.6 × 1.5 cm), and **B** nodule #2 (1.5 × 1.5 cm), were observed in the right upper lung lobe. **C** Nodule #3 (1.5 × 1.5 cm) was observed in the right lower lung lobe. **D**–**F** MWA was performed on nodules #1, #2, and #3 under CT guidance. **G**–**I** A month after MWA, exudative peripheral changes were observed surrounding all three lesions. **J**–**O** Nine months after MWA, the lesions shrank to fibrous cords with no enhancement. The efficacy was assessed to achieve complete ablation

**Fig. 3:**
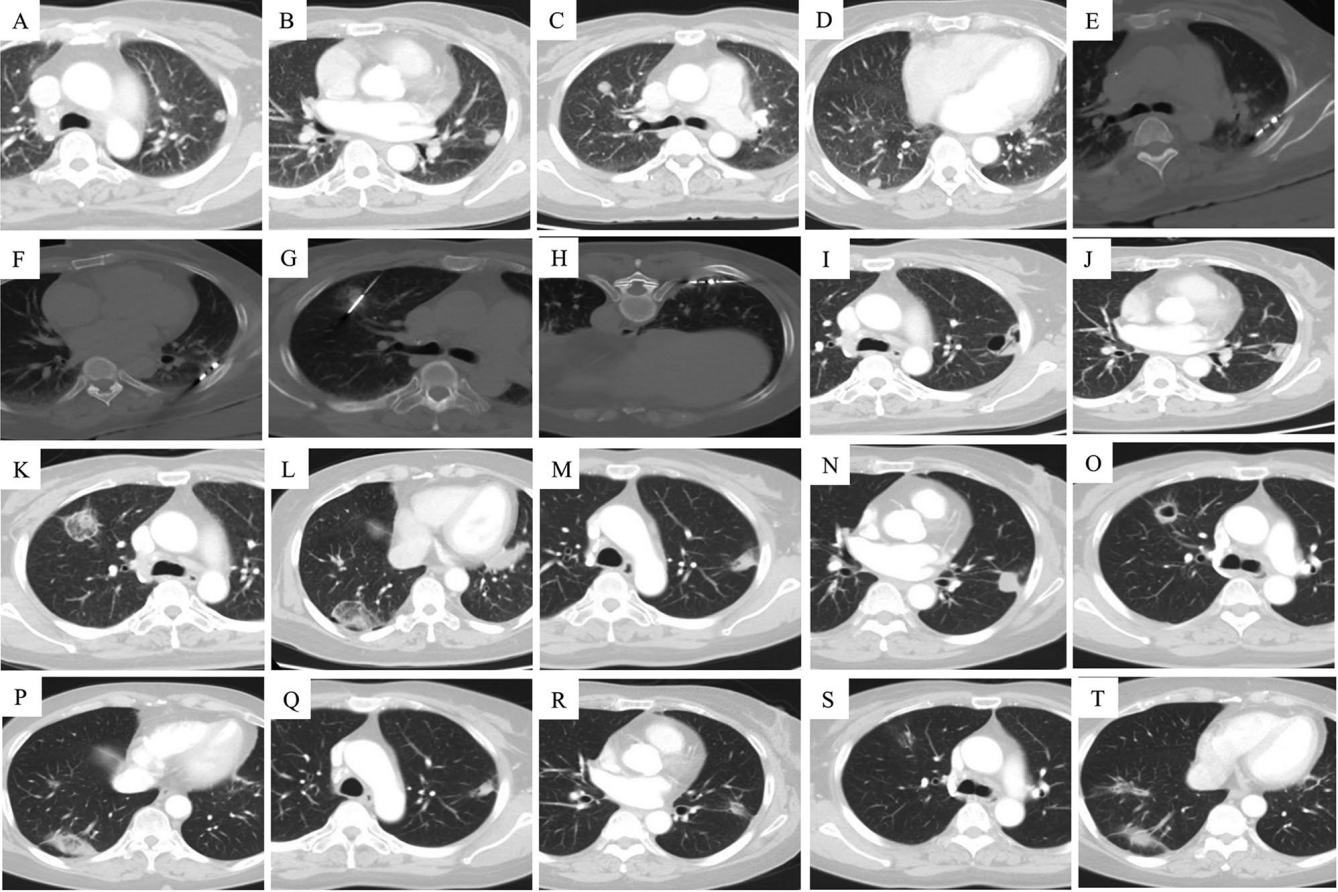
A 63-year-old was found with bilateral lung metastases male 13 months after primary hepatoma carcinoma surgery, and after sorafenib treatment for three months, the lesions’ sizes were enlarged. **A** Nodules #1 (0.9 × 0.8 cm) and **B** Nodule #2 (1.4 × 1.1 cm) were observed in the left upper lung lobe. **C** Nodule #3 (1.4 × 1.1 cm) was observed in the right upper lung lobe. **D** Nodule #4 (1.3 × 1.1 cm) was observed in the right lower lung lobe. **E**, **F** MWA was performed on nodules #1 and #2 under CT guidance. G&H) MWA was performed on nodules #3 and #4 under CT guidance a month after MWA on nodules #1 and #2. **I**, **J** 2 months after the MWA session on nodules #1 and #2. **K**, **L** 1 month after the MWA session on nodules #3 and #4. **M**, **N** 6 months after the MWA session on nodules #1 and #2. **O**, **P** 5 months after the MWA session on nodules #3 and #4. **Q**, **R** 13 months after the MWA session on nodules #1 and #2, the lesions shrank to fibrous cords with no enhancement. **S**, **T** 12 months after the MWA session on nodules #3 and #4, the lesions shrank to fibrous cords with no enhancement. The efficacy was assessed to achieve complete ablation

The median number of metastatic lesions per patient was 2 (range: 1–5). The median maximum diameter of the oligometastases in the unilateral and bilateral pulmonary was 14 mm (range: 4–27 mm) and 18 mm (range: 7–42 mm), respectively. Of the 147 tumors subjected to MWA, 82 and 65 nodules were found in the unilateral and bilateral pulmonary, respectively. Of 116 ablations, 58, 20, and 6 patients underwent one, two, and three sessions, respectively. Figures 1, 2 and 3 show examples of the treatment and follow-up process.

### Technical success and effectiveness

The procedure was technically successful in all 147 tumors. The median follow-up period for the 83 patients was 21.6 months (range: 5.7–87.8 months). Table 3 shows the tumor response during the follow-up. Of the 147 tumors, 121 (82.3%) were completely ablated (Figs. 1, 2 and 3), 8 were classified as incompletely ablated, and 18 were defined as local progression. There was no statistically significant difference between the unilateral and bilateral oligometastases groups (*p* = 0.48).

**Table 3:** Tumor response evaluation after MWA

	Unilateral oligometastases, n (%)	Bilateral oligometastases, n (%)	Total, n (%)	p-Value
Tumor response evaluation	82	65	147	0.48
Complete ablation	67(81.7%)	54(83.1%)	121(82.3%)	
Incomplete ablation	6(7.3%)	2(3.1%)	8(5.4%)	
Local progression	9(11.0%)	9(13.8%)	18(12.2%)	

### Survival outcomes

**Fig. 4:**
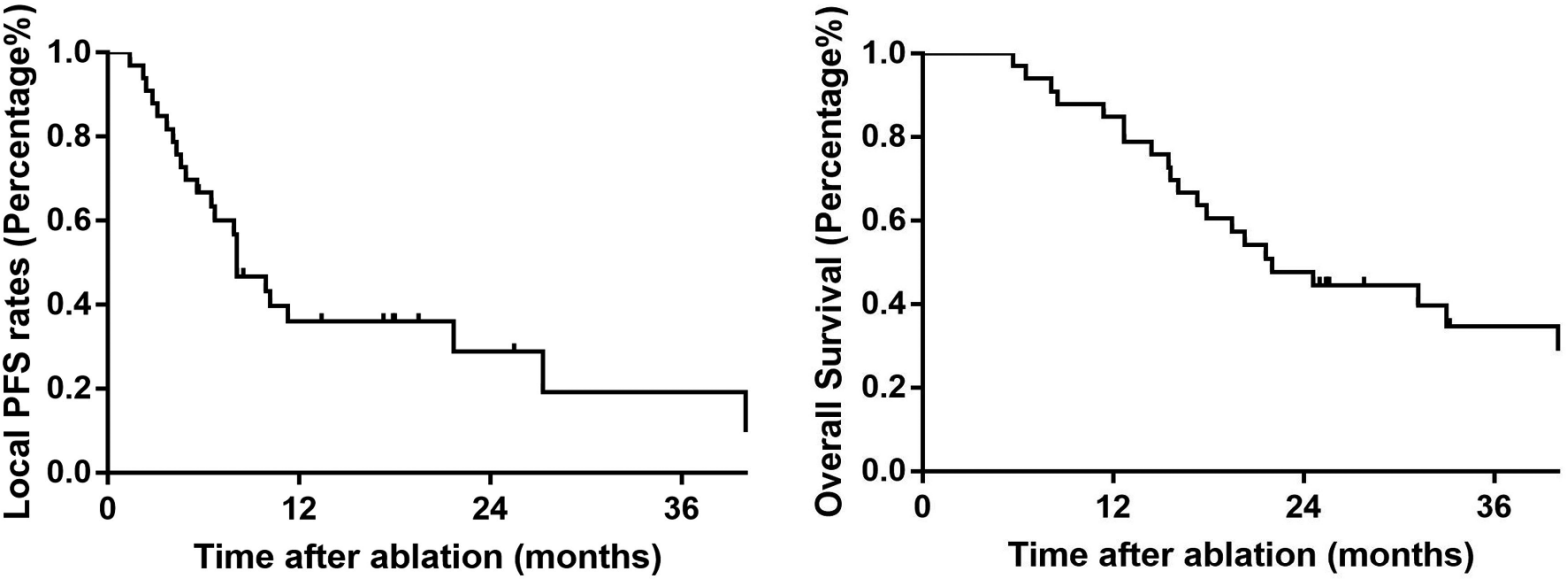
Overall survival and local progression-free survival of patients after ablation

**Fig. 5:**
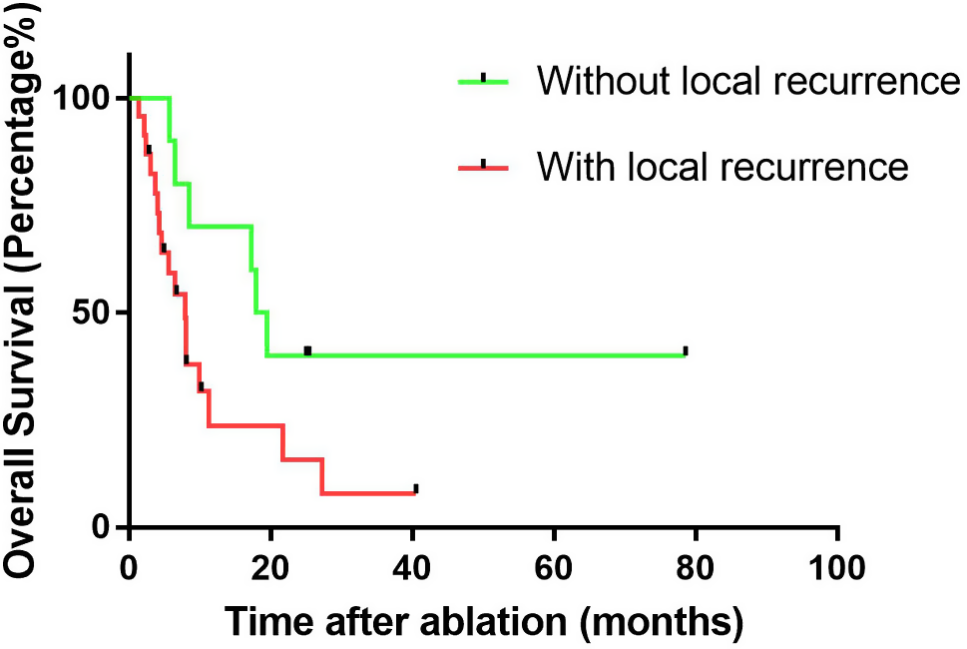
Comparison of the overall survival between patients with and without local recurrence

**Fig. 6:**
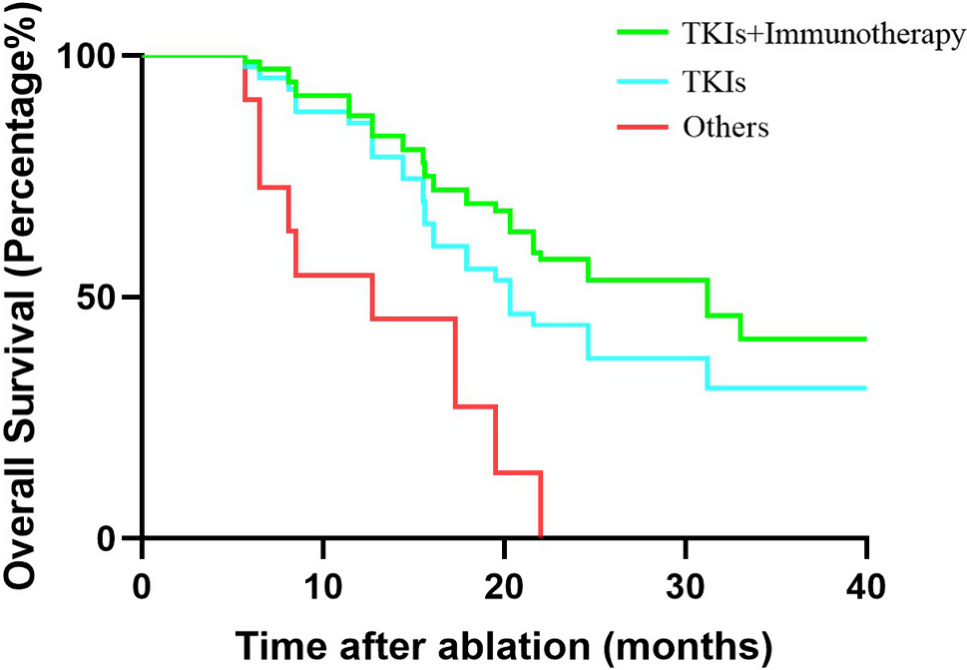
Comparison of the overall survival among patients who received different combinations of treatment

The median follow-up period was 21.6 months (range: 5.7–87.8 months). Figure 4 shows the OS curves of the entire sample. The median OS was 22.0 months. The 1-, 2-, and 3- OS rates were 82.6%, 44.5%, and 25.2%, respectively. The median LPFS was 8.5 months. Then, we compared the OS between patients with and without local progression (Fig. 5). The median survival of the local progression-free group was 18.7 months, while the other group was 7.9 months (*p* = 0.048). Comprehensive treatment including tyrosine kinase inhibitors (TKIs) and/or immunotherapy is essential for HCC patients with distant metastases. For patients enrolled in this trial, before and/or after MWA treatment, most of them received targeted and/or immunotherapy. However, there are still a few patients who refuse the combination treatment because of financial difficulties or potential adverse drug effects. Among these 83 patients, 71 received at least one type of TKI treatment, and 44 patients underwent combined targeted and immunotherapy. In our subsequent analysis, we compared outcomes among the three patient groups (Fig. 6). We observed that the OS for patients receiving combined TKIs and immunotherapy was 31.2 months. In contrast, patients who underwent solely TKI treatment had an OS of 20.3 months, wihle those who declined the combination treatment exhibited an OS of merely 12.7 months. (*p*<0.000).

### Univariate and multivariate Cox proportional hazards regression analyses

Table 4 shows the results of the univariate and multivariate analyses. In the multivariate Cox regression analyses, adjusting for clinically significant univariate factors, AFP levels when oligometastases were initially diagnosed, and the number of oligometastases could serve as independent prognostic factors for OS.

**Table 4:** Univariate and multivariate analyses of prognostic factors

Variation	Univariate		Multivariate	
	HR(95%CI)	P-value		P-Value
Age	1.02(0.98–1.05)	0.376		
AFP levels when oligometastases were diagnosed (ng/mL)	1.00 (1.00–1.00)	0.017	1.00 (1.00–1.00)	0.017
No. of oligometastases	1.29(0.98–1.7)	0.045		
Maximum diameter range of lesion	1.02(0.95–1.08)	0.645		
Sex	0.78(0.23–2.67)	0.695		
Hypertension	0.84(0.25–2.82)	0.773		
Diabetes	0.57(0.13–2.46)	0.455		
Coronary artery disease	1.26(0.29–5.48)	0.757		
Child-Pugh Class	0.72(0.24–2.13)	0.555		
Cirrhosis	1.54(0.60–3.93)	0.365		
Extents of oligometastases	1.46(0.64–3.33)	0.366		

### Complications

Major complications were observed in 12 (12/116, 10.3%) patients, including pneumothorax requiring chest tube placement (4/116; 3.4%) and pleural effusion requiring chest tube drainage (8/116; 6.9%). Minor complications included mild pneumothorax (42/116, 36.2%), pneumonia (10/116,8.6%), mild hemorrhage (10/116,8.6%), and mild pleural effusion (27/116,23.3%). No deaths related to the MWA procedure occurred during the procedure or a month after MWA.

## Discussion

In this study, 100% technical success rate (147/147) was observed for MWA, suggesting that it is technically sound for treating HCC-derived pulmonary oligometastases. Local progression after MWA is a challenging issue for therapeutic management. In this study, 18 out of 142 lesions (12.2%) developed local progression. Of these, 13 recurrent lesions had a maximum diameter of > 3 cm, substantially lower than the tumors with diameters < 3 cm (13 vs. 5). There is no statistically significant difference between the unilateral and bilateral oligometastases groups, which indicates that the lesion size and not the location might be a crucial factor in determining local progression.

Even though metastatic liver cancer is a systemic disorder, reports have shown that local therapies could benefit the prognosis. The prognosis for HCC patients with lung metastasis is usually poor, with a median survival time of only 5.9–8.8 months [18, 19]. However, in our study, the median OS was 22.0 months, indicating that MWAcould effectively improve the prognosis of HCC patients with lung metastasis. Studies have reported that surgical resection can improve the prognosis of patients with lung metastases from HCC. The median survival ranged from 10.7 to 77 months, and the 3-year survival rate was between 26.8% and 75.0% [20]. In this study, the OS and 3-year survival rates of patients treated with MWA were not inferior to surgery. Very few studies have so far compared RFA and MWA in the treatment of lung metastasis by far. A previous study evaluated the therapeutic outcome of percutaneous CT-guided RFA for metastatic pulmonary lesions treatment and reported a higher survival rate and less recurrence rate of MWA than RFA in the first 24 months of follow up, but this conclusion was limited by small sample sizes [21]. Nevertheless, our data indicate that MWA is effective for treating lung metastases in HCC.

We also examined the safety of MWA. No fatalities occurred during or one month after MWA. We observed major complications, including pneumothorax requiring chest tube placement and pleural effusion requiring chest tube drainage, in only 10.3% of the patients, indicating the technique’s safety. The safety of ablation treatment for lung lesions has been studied extensively [22–24]. In a retrospective study, Huang et al. reported that 33 patients with 103 GGOs (mean size = 12.3 mm) underwent 66 ablation procedures with 100% technical success and no MWA procedure-related deaths. The safety was also confirmed with minor and major complications [25]. Several clinical studies have found that ablation treatment is a safer and more effective alternative to surgical resection for treating early-stage malignant tumors [26, 27]. Further, ablation treatment is associated with fewer complications and a shorter hospital stay than surgical resection [24, 28]. These results indicate that MWA is a safe option with a low risk of complications for treating lung tumors.

In this study, the number of oligometastases was an independent prognostic factor for OS. Akhan et al. evaluated the survival benefit after radiofrequency ablation (RFA) treatment of primary and metastatic lung tumors. They found that the tumor status (solitary or multiple) and the presence of extrapulmonary metastasis after the initial RFA of 49 patients who underwent CT-guided percutaneous RFA were significant prognostic factors associated with recurrence-free survival [6]. Li et al. studied the prognostic variables related to survival rate following ablation treatment for pulmonary metastases. They found that patients with < 3 pulmonary metastases showed higher survival rates than those with more than 3 metastases [29]. In addition, AFP levels when oligometastases were diagnosed was an independent prognostic factor, higher AFP levels, which is consistent with previous findings, is associated with a poorer prognosis [30]. We also found that the survival of patients without local recurrence was higher than those with recurrence. Numerous factors contribute to local recurrence post-ablation. Emerging evidence suggests a strong correlation between tumor diameter and recurrence rates following thermal ablation. Specifically, prior research indicates a markedly lower recurrence rate in tumors measuring less than 2 cm in diameter compared to those 2 cm or larger [31]. Additionally, the margin of ablation and the tumor’s anatomical positioning are critical determinants of local recurrence [5, 16]. Furthermore, the histological characteristics of HCC may be implicated in local recurrence rates, poorly differentiated HCC is associated with increased local recurrence post-ablation, which need further validation. Lung metastasis recurrence survival is usually shorter than initial metastasis, probably due to tumor heterogeneity and drug resistance.

This research has certain limitations. Given the retroactive character of this investigation, a selection bias was inevitable, and a prospective, randomized, multicenter-controlled study is still needed. The limited sample size might also skew the conclusions. Moreover, the impact of MWA treatment and other therapies, such as chemotherapy and molecular targeted therapy, on patients’ prognosis needs to be further analyzed.

## Conclusion

In conclusion, MWA might be an effective and safe option for treating HCC-derived pulmonary oligometastases. However, several questions remain unanswered, such as how to combine MWA with other therapeutic approaches to improve patient prognosis and the comparison of the effectiveness of MWA with other treatments.

## Data Availability

The datasets used and/or analyzed during the current study are available from the corresponding author on reasonable request.

## Abbreviations

HCC: hepatocellular carcinoma
MWA: microwave ablation
OS: overall survival
LPFS: local progression-free survival
AFP: α-fetoprotein
IGTA: image-guided thermal ablation
ECOG: Eastern Cooperative Oncology Group
GTR: gross tumor region
GGO: ground-glass opacity
PTZ: post-ablation tumor zone
CT: computed tomography
SIR: American Society of Interventional Radiology
GCP: Good Clinical Practice
BCLC: Barcelona clinic liver cancer
CNLC: China liver cancer
TKI: Tyrosine kinase inhibitors
RFA: radiofrequency ablation
